# USING MODELING TO COMPARE THE ABILITY OF FALLS SCREENING TOOLS TO PREDICT FUTURE FALLS

**DOI:** 10.1093/geroni/igz038.2094

**Published:** 2019-11-08

**Authors:** Elizabeth R Burns, Vicki J Pineau, Rosalind Koff, Sarah Hodge, Bess Welch

**Affiliations:** 1 Centers for Disease Control and Prevention, National Center for Injury Prevention and Control, Division of Unintentional Injury, Atlanta, Georgia, United States; 2 NORC, Bethesda, Maryland, United States; 3 NORC, Chicago, Illinois, United States

## Abstract

The STEADI initiative recommends screening older adults for falls annually using either the 12-item “Stay Independent” or the “three-key questions” screening tools. Both tools ask about falling in the preceding year. However, the comparative predictability of each tool has not been assessed. In response, CDC and NORC, assessed both tools’ ability to predict falls at six and twelve months. Adults 65+ (n=1900), were recruited from a nationally representative panel and were screened for fall risk at baseline using both tools and then followed for a year to determine if they fell. At baseline, 38% of older adults were categorized at-risk of falling based on the 12-item “Stay Independent” and 56% were considered at-risk based on the three-key questions. The history of falling question was excluded for the six month analyses. The “Stay Independent” identified 60% of fallers and the remaining two questions of the three-key questions identified 57% of fallers.

